# Analytical Validation of a Novel Point‐Of‐Care Quantitative Immunoassay for Feline N‐Terminal Pro‐Brain Natriuretic Peptide

**DOI:** 10.1111/vcp.70062

**Published:** 2025-11-04

**Authors:** Emily A. Javery, Ryan C. Fries, Saki Kadotani, Leah Kruckman, Lindsey Humphries, Sumana R. Prabhakar, Michael F. Rosser

**Affiliations:** ^1^ Department of Veterinary Clinical Medicine College of Veterinary Medicine, University of Illinois Urbana‐Champaign Illinois USA

**Keywords:** cardiac biomarker, cat, methods comparisons, point‐of‐care

## Abstract

**Background:**

Point‐of‐care testing (POC) is widely utilized for rapid results for many different analytes. A new feline‐specific N‐terminal pro‐brain natriuretic peptide (NT‐proBNP) quantitative assay (Vcheck V200, Bionote Inc) is currently available but has not undergone independent validation.

**Objective:**

To validate the Vcheck POC quantitative assay for feline NT‐proBNP.

**Methods:**

Validation was performed in accordance with the American Society for Veterinary Clinical Pathology guidelines utilizing serum samples from 62 cats. Precision was determined for low (50–100 pmol/L), mid (101–300 pmol/L), and high (> 301 pmol/L) pools short‐term (20 repetitions) and long‐term (5 repetitions each day for 5 days). Linearity, methods comparison, interference testing, and sample stability were evaluated.

**Results:**

The within‐day coefficients of variability (CV) were low pool = 12.6%, mid pool = 10.4%, and high pool = 8.7%. The within‐week CV was low pool = 9.9%, mid pool = 14.9%, and high pool = 6.9%. The assay was linear over the analytical range of 53–1488 pmol/L (*R* ^2^ = 0.99, *p* < 0.0001). Paired samples between the feline Cardiopet NT‐proBNP (IDEXX) and Vcheck assays demonstrated a mean difference of 11 pmol/L (2.3%), *p* = 0.38, between assays with minimal constant or proportional bias. Hemolysis and lipemia did not affect assay performance, while all icteric samples were invalid. Significantly lower values were identified in samples after 2 and 4 h when stored at 20°C and 4°C, respectively.

**Conclusions:**

The Vcheck V200 has acceptable precision, accuracy, and compares favorably with commercially available assays and is a viable POC quantitative assay for feline NT‐proBNP.

## 1 Introduction

Brain‐type natriuretic peptide (BNP) is a hormone released by cardiomyocytes, resulting in natriuresis, diuresis, and vasodilation [1]. The secretion of BNP is significantly upregulated in cardiac disease and failure in response to myocyte stress and stretch from increased volume and pressure [2]. BNP is secreted as a prohormone (proBNP) and is cleaved by furin into the active BNP hormone and inactive N‐terminal pro B‐type natriuretic peptide (NT‐proBNP) [1]. NT‐proBNP has a significantly longer half‐life than BNP or proBNP and thus is a better diagnostic marker to assess the magnitude of myocardial wall stress or stretch [3].

In feline patients, quantitative NT‐proBNP has been utilized in the clinical setting to differentiate between cardiac and pulmonary disease for patients presenting with respiratory signs and to increase the likelihood of identifying patients with moderate to severe preclinical or occult cardiac disease [4, 5, 6, 7]. Several studies have investigated the ability of NT‐proBNP to identify cats with subclinical disease and have determined that a cutoff of > 100 pmol/L yields the most clinically useful results [5]. Furthermore, an NT‐proBNP concentration > 270 pmol/L can accurately distinguish between cardiogenic and noncardiogenic causes of respiratory clinical signs [8]. Until recently, quantitative analysis required shipping to a commercial facility for testing, rendering samples subject to degradation and preanalytical error. Results may also take up to 72 h to return, which could delay diagnosis and initiation of proper treatment. While a point‐of‐care qualitative NT‐proBNP colorimetric ELISA assay (IDEXX, Westbrook, ME, USA) is available for feline patients, because of low positive predictive values and specificity, quantitative assessment is considered ideal [9].

A new quantitative NT‐proBNP assay (Vcheck V200, Bionote Inc) is currently available and has been validated for use in canine serum [10]. We hypothesized that the Vcheck NT‐proBNP assay would accurately measure NT‐proBNP in feline serum. The objectives of this study were to evaluate the performance of the Vcheck assay for the measurement of NT‐proBNP in feline serum, assess the effects of time and temperature on the degradation of feline NT‐proBNP in serum, and establish an upper reference for serum NT‐proBNP using the Vcheck assay in healthy cats.

## 2 Materials and Methods

### 2.1 Sample Collection, Handling, and Storage

1. Collection: serum samples from surplus feline blood submitted for testing to the University of Illinois Clinical Pathology Laboratory and fresh samples from client‐owned cats evaluated at the University of Illinois Veterinary Teaching Hospital were utilized. This study was approved by the institutional animal care and use committee of the University of Illinois at Urbana‐Champaign (ID: 21157). Owner consent was obtained for all cats enrolled. For fresh samples, whole blood was collected via venipuncture of the external jugular or medial saphenous vein and placed into plain serum tubes without a serum separator. Samples were allowed to clot for at least 15 min and then centrifuged at 20°C at 14 800 rpm for 10 min using an E8 Digital Combination Centrifuge with Crit Carrier (LW Scientific) and the serum was separated from the packed cells. Any fresh samples not analyzed within 2‐h were stored at −80°C for analytical performance testing (see Section 2.3). Surplus samples were obtained by convenience independent of underlying disease process and were used exclusively as a diluent as all samples had NT‐proBNP levels below the detectable range of the assay (< 50 pmol/L). For these samples, serum was frozen at −20°C within 12 h of collection and stored for at least 7 days. After this time, the samples were thawed for 10 min at 20°C to allow for pooling prior to storing at −80°C. Samples collected for mid pool and high pool were obtained from cats with primary cardiomyopathy diagnosed by a board‐certified cardiologist using echocardiography. All samples used in this study were subjectively macroscopically clear of hemolysis, lipemia, and icterus.
2. Precision, Linearity, Interference: serum samples were pooled to obtain clinically relevant target concentrations of low (50–100 pmol/L), mid (101–300 pmol/L), and high (> 301 pmol/L), rather than the reportable assay range, and they were stored at −80°C up to 6 months until analysis. NT‐proBNP concentrations for each sample were established using the Cardiopet NT‐proBNP (IDEXX) assay and target pool concentrations were established using the Vcheck assay.

### 2.2 Quantitative Measurement of NT‐proBNP

Quantitative measurement of NT‐proBNP was performed using the Vcheck 200 (Bionote) analyzer or the Cardiopet NT‐proBNP (IDEXX) assay. The Vcheck 200 uses anti‐feline NT‐proBNP antibodies with conjugated fluorescence microparticles. The Vcheck 200 was calibrated prior to use, and every 30 days, using Bionote's recommended software and calibration cartridges performed in accordance with the manufacturer's instructions. There are no available assayed quality control materials with a concentration in the linear range of the feline assay. Once calibrated, 100 μL of serum was placed in the supplied diluent tube and mixed by pipetting. The mixture was transferred to the well on the feline NT‐proBNP dry chemical cartridge prior to inserting it into the Vcheck 200. Results were reported after a 10‐min incubation. The assay has a reportable range of 50–1500 pmol/L, set by the manufacturer, and any concentrations outside of this range are reported as < 50 pmol/L or > 1500 pmol/L, respectively. The feline Cardiopet NT‐proBNP (IDEXX) is a second‐generation sandwich ELISA containing unique anti‐feline NT‐proBNP capture and detection antibodies. The assay is reportedly validated for use in feline serum and plasma with a reportable range of 24–1500 pmol/L, and any concentrations outside of this range are reported as < 24 pmol/L or > 1500 pmol/L, respectively [11].

### 2.3 Analytical Performance of NT‐proBNP Vcheck 200 Analyzer

Precision (short‐term and long‐term replication), linearity, methods comparison, and interference were evaluated in accordance with the ASVCP quality assurance guidelines [12]. Recovery was not evaluated as a feline NT‐proBNP standard solution was not commercially available at the time of this study. The detection limit was not independently determined, as low values are not clinically significant for this assay. QC validation was not feasible as commercial control products within the relevant range were not available.

1. Precision within the manufacturer's reportable range was determined for the targeted low (50–100 pmol/L), mid (101–300 pmol/L), and high (> 301 pmol/L) concentration pools within‐day, over the course of approximately 6 h (short‐term, 20 repetitions) and long‐term (inter‐assay, 5 repetitions each day for 5 consecutive days). Because samples were aliquoted prior to freezing at −80°C, individual aliquots were thawed at 20°C each morning prior to analysis and stored at 4°C throughout the day. Precision analysis below the manufacturer's lower limit of quantification (50 pmol/L) was investigated with 20 replicate serum samples with a target concentration of < 50 pmol/L as measured by the Vcheck analyzer that had a measured concentration of approximately 37 pmol/L using the Cardiopet NT‐proBNP assay. Precision analysis above the manufacturer's upper limit of quantification was investigated with 20 replicate serum samples with a target concentration of > 1500 pmol/L, as measured by the Vcheck analyzer, which had a measured concentration of > 1500 pmol/L using the Cardiopet NT‐proBNP assay.
2. Linearity of the assay was determined by using serial dilutions of pooled serum. The serum serial dilutions were made by mixing pooled serum with high NT‐proBNP concentrations nearing the manufacturer's upper limit of quantification (1488 pmol/L) into pooled serum with NT‐proBNP values below the reportable range of the test. All samples had been used previously and refrozen at −80°C for 1 month. The pooled serum sample below the reportable range was used as the diluent to maintain a constant feline serum matrix and tested in triplicate to confirm that it had a less than detectable value. Ten 100 μL test samples were made by mixing increasing volumes of high NT‐proBNP concentration pooled serum in approximately 10% increments with decreasing volumes of below the reportable range pooled serum. All test samples were measured in triplicate.
3. Method comparison was determined from paired serum samples (*n* = 49). Paired serum samples were stored at −80°C for up to 6 months. One set of paired samples was submitted to IDEXX Laboratories for analysis using the Cardiopet NT‐proBNP assay and the other set of samples using the Vcheck analyzer. Samples submitted to IDEXX were transferred from a −80°C freezer and shipped on dry ice, expedited overnight and all samples were confirmed to be frozen upon receipt. All samples were analyzed in duplicate during the same 3 days.
4. Effects of test interference from hemolysis, icterus, and lipemia (turbidity) were evaluated. Hemolytic, icteric, and lipemic indices were measured for a pooled serum sample obtained from cats with cardiac disease on an AU680 chemistry analyzer (Beckman Coulter, Brea, California) and confirmed to be negative for all interfering substances prior to testing. The NT‐proBNP concentration of the pooled serum was determined in duplicate to determine a baseline value.
  Multiple samples of discarded feline K + EDTA blood were used to form a hemolysis stock solution. Samples had been stored at 4°C for 3–4 days prior to use and were centrifuged for 10 min at 3500 rpm. Plasma was discarded, and the remaining cell pellet was washed three times in 0.9% NaCl. After the final wash, the supernatant was discarded and replaced with 1.5 mL deionized water. The resulting solution was transferred to a polypropylene tube and frozen overnight at −20°C. Following thawing at room temperature the next morning, the sample was centrifuged at 14800 rpm with the E8 Digital Combination Centrifuge with Crit Carrier (LW Scientific) to separate erythrocyte membranes. The resulting supernatant was transferred to a new tube and combined with incremental concentrations of 0.9% NaCl to produce four solutions which, when diluted 1:10 with additional NaCl, were confirmed to give a hemolytic index of 1+ (50–99 mg/dL hemoglobin), 2+ (100–199 mg/dL hemoglobin), 3+ (200–299 mg/dL hemoglobin), and 4+ (> 300 mg/dL hemoglobin) on the AU680 chemistry analyzer. Hemolysis stock solutions were then diluted 1:10 with pooled serum to measure NT‐proBNP in duplicate for each level.
  Lipemia was indirectly assessed using Intralipid 20% (Sigma‐Aldrich), and icterus was assessed using conjugated bilirubin (EMO Millipore, Darmstadt, Germany). Both products were diluted with 0.9% NaCl to produce respective lipemic or icteric indices of 1+ (40–99 mg/dL lipid, 2.5–4.9 mg/dL bilirubin), 2+ (100–199 mg/dL lipid, 5.0–9.9 mg/dL bilirubin), 3+ (200–299 mg/dL lipid, 10.0–19.9 mg/dL bilirubin), and 4+ (> 300 mg/dL lipid, > 20 mg/dL bilirubin) on the AU680 chemistry analyzer, which were generated using a previously described protocol [13]. These stock solutions were diluted 1:10 with pooled feline serum to measure NT‐proBNP in duplicate for each level of each interferent.

### 2.4 Sample Stability

Possible effect of storage was evaluated using fresh feline serum from 10 cats with known cardiac disease and an NT‐proBNP between 800 and 1400 pmol/L. Whole blood was collected via venipuncture of the medial saphenous vein and placed into red top serum tubes without a serum separator. Samples were allowed to clot for 15 min and then centrifuged at 20°C at 14 800 rpm for 10 min, and the serum was separated from the packed cells and baseline tested on the assay (*T* = 0), then held at either room temperature (20°C) or refrigeration (4°C). The refrigerated samples were individually aliquoted into 150 μL tubes and removed only once prior to running the assay. Quantitative NT‐proBNP was evaluated using the Vcheck assay every hour for 6 h and again after 24 h.

### 2.5 Reference Intervals

Fresh serum samples were acquired from continuously recruited clinically healthy cats presenting to the cardiology service at the University of Illinois over 3 months. Samples were collected as described in Section 2.1, and quantitative NT‐proBNP was evaluated using the Vcheck assay within 2 h of collection. Cats were judged to be clinically healthy based on physical examination, complete blood count, serum biochemistry including total T4, indirect Doppler blood pressure, and echocardiogram.

### 2.6 Statistical Analysis

All statistical analysis was performed using commercially available software (GraphPad Prism Software Version 10.0.0, Boston, MA, USA, and MedCalc Software Version 20.211, Ostend, Belgium). Coefficients of variation (CV) were determined from short‐term (20 within‐day) replicates and long‐term (25 within‐week) replicates for low, mid, and high concentration pools. Acceptable analytical imprecision was considered as less than half of the within‐subject biological variability of feline NT‐proBNP in healthy cats for low‐pool samples and occult cardiomyopathy cats for mid‐ and high‐pool samples. The weekly within‐subject CV for NT‐proBNP in healthy cats has been reported as 21.2% and 28.1%, respectively, and can exceed 65% in some individuals [14, 15]. The weekly within‐subject CV for NT‐proBNP in occult hypertrophic cardiomyopathy cats is reportedly 29% [15]. Linearity of the assay was determined by simple linear regression. Assays were compared using Passing‐Bablok regression and Bland–Altman plots. Sample stability was evaluated using repeated measures ANOVA with Dunnett's multiple comparisons test. Interference was analyzed using a paired *t*‐test and repeated measures ANOVA with Dunnett's multiple comparisons test, with *p* < 0.05 considered statistically significant. During methods comparisons, any samples below or above the analytical range of the Vcheck assay (50 and 1500 pmol/L) were assigned values of 49 and 1501 pmol/L, respectively. Reference intervals were generated using nonparametric percentile methods following the American Society of Veterinary Clinical Pathology guidelines [16]. A *p* < 0.05 was considered statistically significant.

## 3 Results

### 3.1 Analytical Performance

**FIGURE 1:**
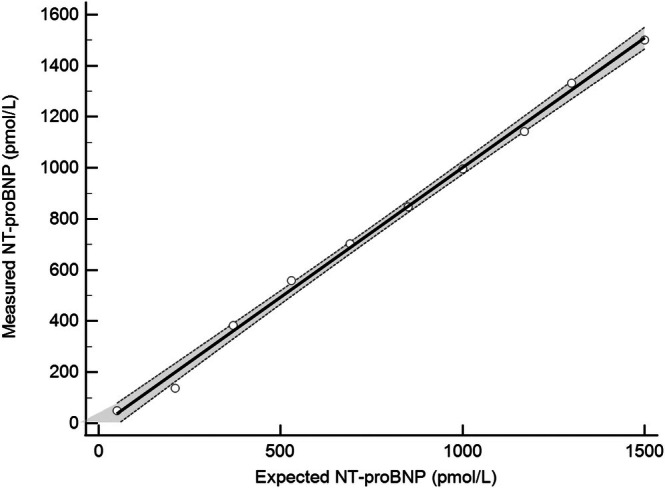
Linear assessment using triplicate measurement of pooled serum with high NT‐proBNP concentration serially diluted ten times, spanning the reportable range of the analyzer. The assay is linear across the range 53–1488 pmol/L (*y* = 1.01X − 12.3). The solid black line represents the ordinary linear regression line with the 95% CI (dashed lines). The open circles represent the mean value of the triplicate measurements at each dilution. NT‐proBNP, N‐terminal pro B‐type natriuretic peptide.

**FIGURE 2:**
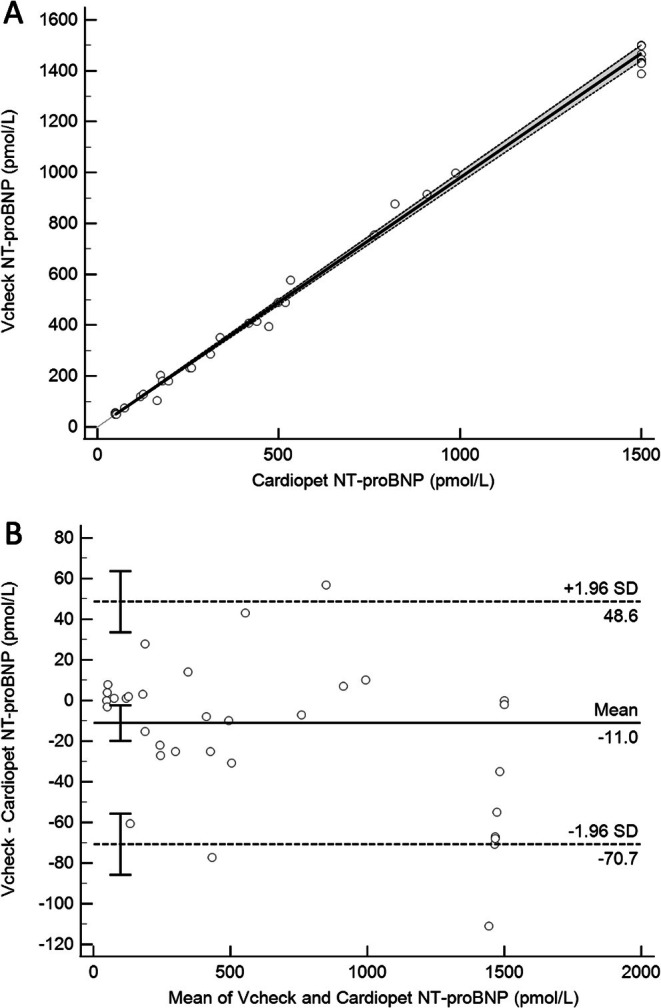
(A) Scatterplot comparison of the Vcheck and Cardiopet NT‐proBNP assays using Passing‐Bablok regression for 49 patient samples showing excellent agreement (*y* = 1.03 + 0.98X). The thin diagonal line is the line of identity, the thicker black line is the line of best fit from Passing‐Bablok regression, and the dashed lines indicate the 95% confidence interval. (B) Bland–Altman plot of the difference between NT‐proBNP concentration measured in paired serum samples by the Vcheck assay compared to the Cardiopet NT‐proBNP assay, showing a mild negative bias of 11 pmol/L for the Vcheck assay. The solid line is the mean of the differences, and the two dashed lines represent the 95% limits of agreement. NT‐proBNP, N‐terminal pro B‐type natriuretic peptide.

1. The analysis of imprecision showed that CVs varied across concentrations, with the lowest CV at the higher concentrations. All CVs were < 15% and were lower for the long‐term samples, compared with the short‐term, for the low and high concentrations (Table 1). All CVs were < 50% of the reported within‐subject CV, except for the short‐term low pool when assessed using a within‐subject CV of 21.2%. This CV was, however, < 50% when assessed using a within‐subject CV of 28.1%. Of the 20 replicates below the reportable range, all values were correctly reported as < 50 pmol/L using the Vcheck NT‐proBNP assay. Of the 20 replicates above the reportable range, 14 values of > 1500 pmol/L were reported correctly, with the 6 remaining samples ranging between 1231 and 1455 pmol/L. Without a gold standard method for feline NT‐proBNP or an available QCM within the linear range, bias for the purposes of total error calculation could not be adequately determined. Therefore, the total error was estimated using 2 × CV. Based on this study, the expected total observed error in the Vcheck NT‐proBNP assay will range from approximately 18%–25% at concentrations < 100 pmol/L and 14%–17% at concentrations > 300 pmol/L, with expected continued linear decreases in CV, and therefore total error, at higher concentrations [10].
2. After serial dilutions, linearity of the assay was evaluated from measured NT‐proBNP concentrations ranging from 53 to 1488 pmol/L spanning nearly the entire analytical range reported by the manufacturer (50–1500 pmol/L). Regression analysis showed a linear relationship, *y* = 1.01X − 12.3 (*R* ^2^ = 0.99, *p* < 0.0001) (Figure 1). The 95% CI of the y‐intercept for the regression line (−56.5, 31.8) included 0, and the 95% CI of the slope for the regression line (0.96, 1.06) included 1.
3. When comparing the VCheck NT‐proBNP assay to the Cardiopet NT‐proBNP assay, results of Passing‐Bablok regression indicate minimal bias between assays, with a y‐intercept of 1.03 pmol/L and a 95% CI (0, 1.95) that included 0. There was minimal proportional bias based on a slope of 0.98 with a 95% CI (0.96, 1.00) that includes 1 (Figure 2A). Visual inspection of the data using the Bland–Altman revealed a mean difference of −11.0 pmol/L (95% CI −17.8 pmol/L—1.3 pmol/L), or −2.3% when comparing the Vcheck NT‐proBNP assay to the Cardiopet NT‐proBNP assay (Figure 2B) which is not significant (*p* = 0.38).
4. The analysis of interference from hemolysis, icterus, and lipemia (turbidity) was performed on pooled samples with an NT‐proBNP concentration of 329 pmol/L for hemolysis and 543 pmol/L for icterus and lipemia. There was no significant interference observed for any levels of hemolysis or lipemia; all P values were > 0.05, and the percent change from the neat NT‐proBNP concentration was less than 10%. All values for all icteric samples were reported as an invalid code on the analyzer (Table 2) precluding statistical analysis.

**TABLE 1:** Short‐term and long‐term precision of the Vcheck feline NT‐proBNP assay across low, mid, and high concentrations.

	Short‐term			Long‐term		
	NT‐proBNP (pmol/L)	CV (%)	SD	NT‐proBNP (pmol/L)	CV (%)	SD
Low	86.8	12.6	8.9	88.4	9.9	8.4
Mid	102	10.4	3.6	200.3	14.9	6.5
High	392.4	8.7	4.9	361	6.9	7.1

*Note:* The NT‐proBNP values represent the mean measurement for each target pool.

Abbreviations: CV, coefficient of variation; NT‐proBNP, N‐terminal pro‐brain natriuretic peptide; SD, standard deviation.

**TABLE 2:** Results of interference analysis for hemolysis, lipemia, and icterus.

NT‐proBNP concentration pmol/L	NT‐proBNP concentration, pmol/L (% change from neat)	NT‐proBNP concentration, pmol/L (% change from neat)	NT‐proBNP concentration, pmol/L (% change from neat)	NT‐proBNP concentration, pmol/L (% change from neat)	Mean O/E ± SD (%)
Neat	Hemolysis 1+ (50–99 mg/dL hemoglobin)	Hemolysis 2+ (100–199 mg/dL hemoglobin)	Hemolysis 3+ (200–299 mg/dL hemoglobin)	Hemolysis 4+ (> 300 mg/dL hemoglobin)	
329	334 (+1.5%) *p* = 0.14	347 (+5.5%) *p* = 0.22	359 (+9.1%) *p* = 0.08	339 (+3.0%) *p* = 0.21	105 ± 3.0
Neat	Lipemia 1+ (40‐99 mg/dL intralipid)	Lipemia 2+ (100–199 mg/dL intralipid)	Lipemia 3+ (200–299 mg/dL intralipid)	Lipemia 4+ (> 300 mg/dL intralipid)	Mean O/E ±SD (%)
543	549 (+1.1%) *p* = 0.90	489 (−9.9%) *p* = 0.47	505 (−7.0%) *p* = 0.68	564 (+3.9%) *p* = 0.76	97 ± 6.5
Neat	Icterus 1+ (2.5–4.9 mg/dL bilirubin)	Icterus 2+ (5.0–9.9 mg/dL bilirubin)	Icterus 3+ (10.0–19.9 mg/dL bilirubin)	Icterus 4+ (> 20 mg/dL bilirubin)	Mean O/E ±SD (%)
543	Invalid	Invalid	Invalid	Invalid	Invalid

*Note:* All NT‐proBNP concentrations are reported as the mean or invalid when a quantitative value could not be determined. The measured NT‐proBNP concentration and the percent difference are provided. The *p* value for all comparisons was > 0.05.

Abbreviations: NT‐proBNP, N‐terminal pro‐brain natriuretic peptide; O/E, observed/expected.

### 3.2 Sample Stability

**FIGURE 3:**
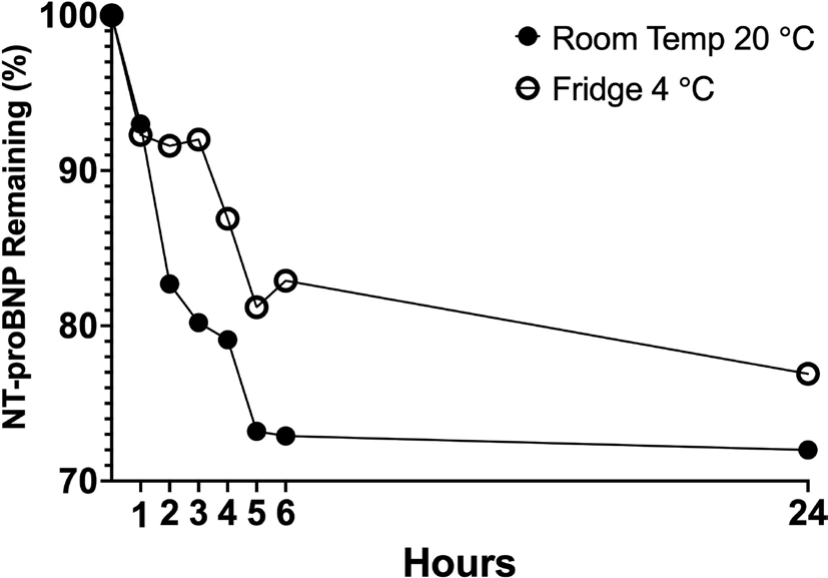
An XY graph displaying the % of NT‐proBNP concentration in 10 paired fresh serum samples held under refrigeration or room temperature over time. Samples were analyzed at hours 0, 1, 2, 3, 4, 5, 6, and 24. Room temperature samples were > 10% and significantly different from baseline after 2 h (−17.3%, *p* = 0.003), and refrigerated samples were > 10% and significantly different after 4 h (−14.1%, *p* = 0.019).

The mean percent recovery over the first 6 h and at 24 h is displayed in Figure 3. The mean baseline (time 0) NT‐proBNP concentration for all samples was 1075 pmol/L. Repeated measures ANOVA with Dunnett's multiple comparisons test determined that at room temperature, after 2 h, samples were significantly different from baseline with an 82.1% recovery and a mean difference of 185.2 pmol/L (*p* = 0.003). Under refrigerated conditions, samples were significantly different from baseline after 4 h with an 86.9% recovery and a mean difference of 140 pmol/L (*p* = 0.019). While the concentration of NT‐proBNP declined in all samples, the rate of decline was faster at room temperature. Comparison of room temperature to refrigeration determined that samples were significantly different at the 2‐h (*p* = 0.009) and 3‐h (*p* = 0.014) time points, but no longer different after 4 h.

### 3.3 Reference Intervals

**FIGURE 4:**
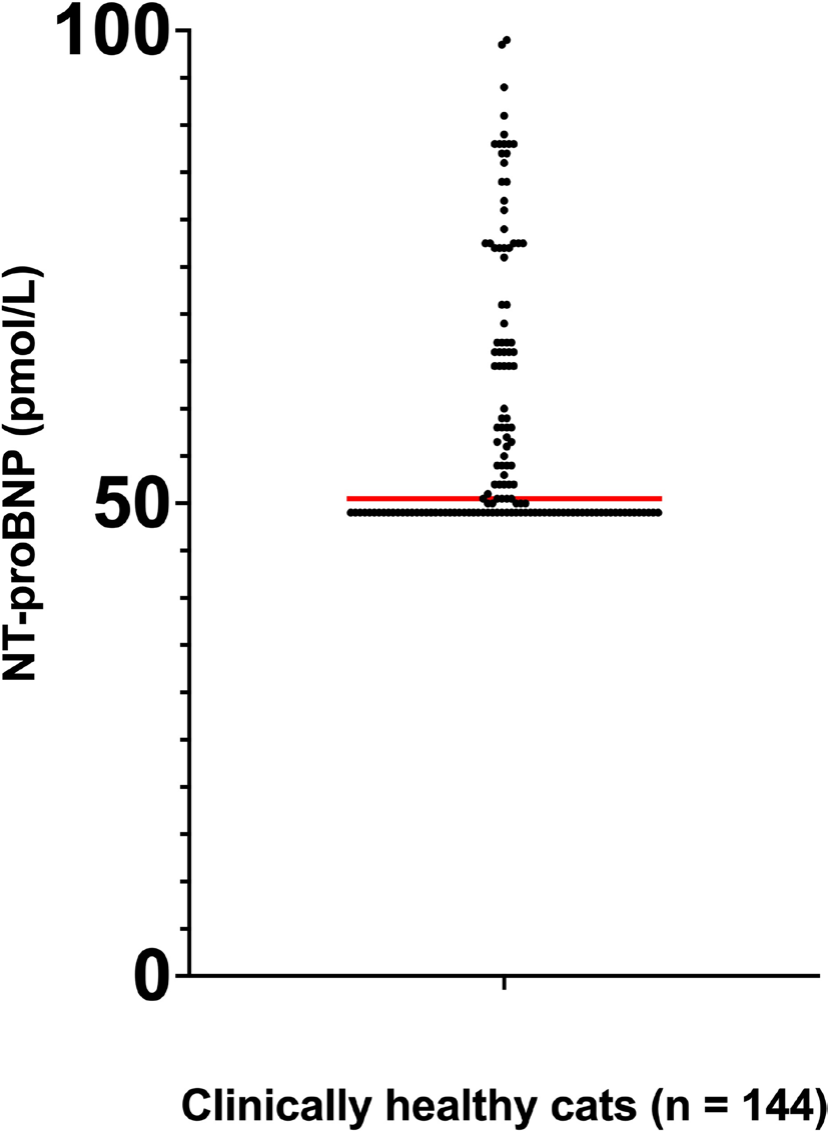
Distribution of the serum NT‐proBNP concentrations generated using the Vcheck assay. The reference population consisted of 144 apparently healthy cats with a normal echocardiogram. The red line denoted the median of the NT‐proBNP concentrations.

This population consisted of 144 healthy cats (male = 83, female = 61) with a median age of 5 years (range 1–15 years) and a mean age of 6.2 ± 3.6 years. Breed was not reported for all cats. The distribution of NT‐proBNP was nonparametric (*p* < 0.003) based on the Shapiro‐Francia test. Tukey's outlier analysis was performed, and no outliers were identified or removed. The nonparametric percentile method was used to establish the 95th percentile reference interval with 90% confidence intervals. The median NT‐proBNP concentration in serum was 50.5 pmol/L (minimum = < 50 pmol/L, maximum = 99 pmol/L, mean = 58.8 pmol/L; Figure 4). The upper limit of the reference interval for the Vcheck NT‐proBNP assay was 92.1 pmol/L based on the 95th percentile with a 90% CI around the upper limit of 88–99 pmol/L in these 144 healthy cats. While the lower limit of the reference interval for the VCheck NT‐proBNP assay was < 50 pmol/L based on the 95th percentile, with a 90% CI around the lower limit of < 50 to < 50 pmol/L in these 144 healthy cats.

## 4 Discussion

The Vcheck feline NT‐proBNP assay is an acceptable point‐of‐care test for quantitative use in feline serum. In our study, the Vcheck NT‐proBNP assay demonstrated acceptable precision, linearity, and minimal bias when compared with the Cardiopet NT‐proBNP assay available through a commercial laboratory.

The CVs for the Vcheck NT‐proBNP assay were less than 15% for all concentration pools, both short‐term and long‐term. Levels of imprecision were comparable to those described in a study evaluating the same analyzer for use in dogs, with higher imprecision at the lower end of the reportable range as expected for an immunoassay [12]. The ASVCP has not assigned a total allowable error (TEa) to NT‐proBNP. Similar to NT‐proBNP, troponin I is another cardiac biomarker that can be measured by immunoassay and was assigned a 70% TEa with a maximal CV of 20% by the ASVCP Quality and Laboratory Assurance Committee [17]. Additionally, there are two studies that report the biologic variability of NT‐proBNP in healthy and hypertrophic cardiomyopathy cats. The weekly within‐subject CV for NT‐proBNP in healthy cats has been reported as 21.2% and 28.1%, respectively, and can exceed 65% in some individuals [14, 15]. The weekly within‐subject CV for NT‐proBNP in occult hypertrophic cardiomyopathy cats is reportedly 29% [15]. The Vcheck NT‐proBNP assay had CVs that exceeded our quality goal of less than half of the within‐subject CV at all concentrations, with the exception of the within‐day low‐pool CV of 12.6%. Based on the two references, this either represents 59.4% or 44.8% of the within‐subject CV. This could have an impact on serially monitoring cats with NT‐proBNP concentrations < 100 pmol/L using this assay. However, because of the extreme individual variability of NT‐proBNP in healthy cats, it is difficult to determine an accurate within‐subject CV. Importantly, concentrations of NT‐proBNP in healthy cats must increase or decrease by at least 60%–103% to be considered clinically significant, and therefore, the imprecision of the Vcheck assay at low‐pool concentrations appears acceptable [14, 15, 16, 17].

Quantitative NT‐proBNP values, prior to the development of the Vcheck assay, required submission to a commercial laboratory for testing and are subject to pre‐analytical errors and sample degradation due to shipping and processing times. Degradation of NT‐proBNP or pre‐analytical errors could result in falsely lower results and inaccurate diagnosis. To avoid NT‐proBNP degradation > 10%, which could affect clinical interpretation, samples should ideally be centrifuged immediately after clotting and the serum run on the Vcheck assay. Based on the data in this study, room temperature samples decreased by approximately 18% within 2 h of collection, and refrigerated samples decreased by approximately 14% within 4 h. Finally, the Vcheck assay appears to perform adequately in the presence of up to 4+ hemolysis and lipemia; however, no quantitative results could be obtained for any degree of icterus, and therefore icteric samples should not be used. Although the relationship was nonlinear and changes did not reach statistical significance, a consistent positive percent difference was noted in hemolyzed samples. In theory, this could cause misclassification of samples very close to clinical decision thresholds, and interpretation of borderline results from grossly hemolyzed samples should be done with caution.

This study had several limitations, including a lack of a commercially available feline NT‐proBNP standard to aid in the calculation of total observed error or allow for a recovery study. Additionally, in a recent manuscript validating the Vcheck canine NT‐proBNP assay, prospective comparison of fresh samples immediately analyzed on the Vcheck with paired samples shipped for evaluation by the same reference analyzer showed a significant positive bias, with approximately 15% of shipped samples resulting in false negative interpretation [12]. Our methods comparison involved nearly identical storage conditions between methods and optimal transport, and we did not specifically compare fresh in‐clinic NT‐proBNP concentrations on the Vcheck assay to standardly shipped samples to a reference lab. Therefore, we cannot comment on the bias between an immediately run in‐clinic sample and one that is shipped to a commercial laboratory under standard shipping conditions. An additional limitation was the evaluation of only 3 concentration pools, and therefore, we cannot comment on how the assay performs at concentrations significantly higher than 270 pmol/L. Our concentration pools were selected based on the most useful clinical cutoffs described in cats; < 100 pmol/L is considered normal, > 100 pmol/L is optimal for detecting occult cardiac disease, and > 270 pmol/L is optimal for differentiating heart failure from non‐heart failure causes of respiratory distress [5, 8]. It was therefore most important that the performance of the assay be determined around these cutoffs. Furthermore, there is currently no clinical application for NT‐proBNP concentrations at the higher end of the assay range. We assessed interference on high pool samples only and therefore cannot comment on the effects of hemolysis and lipemia on the low pool and mid pool concentrations. Finally, we only assessed the performance of this assay in feline serum as the manufacturer recommends using serum. Therefore, we cannot comment on sample stability or how this assay will perform using other types of samples, such as plasma or pleural effusion.

In conclusion, Vcheck feline NT‐proBNP assay provides acceptable results in a point‐of‐care setting and compares favorably with commercially available assays. Stability and sample quality must be considered when analyzing samples. All samples should be free of any visible icterus and should be utilized or refrigerated immediately and used within 4 h.

## Disclosure

Vcheck V200 analyzer and test cartridges provided by Bionote USA Inc., Big Lake, MN, USA.

## Conflicts of Interest

Bionote USA Inc., Big Lake, MN, USA, providing the instrumentation and assays for method validation.
